# Dielectrowetting Control of Capillary Force (Cheerios Effect) between Floating Objects and Wall for Dielectric Fluid

**DOI:** 10.3390/mi12030341

**Published:** 2021-03-23

**Authors:** Junqi Yuan, Jian Feng, Sung Kwon Cho

**Affiliations:** Department of Mechanical Engineering and Materials Science, University of Pittsburgh, Pittsburgh, PA 15237, USA; juy16@pitt.edu (J.Y.); jif17@pitt.edu (J.F.)

**Keywords:** surface tension, electro-capillary, microfluidics

## Abstract

A capillary interaction between floating objects and adjacent walls, which is known as “Cheerios effect”, is a common phenomenon that generates capillary attraction or repulsion forces between them depending on their wettabilities, densities, geometries, and so on. This paper deals with controlling the capillary forces, specifically, acting on objects floating on a dielectric (non-conductive) fluid. A key control input parameter is the wettability (contact angle) of the sidewall adjacent to the floating object. By introducing dielectrowetting to the sidewall and actively changing the contact angle on the sidewall, the capillary force is controlled and easily reversed between attraction and repulsion. In this reversing process, the tilting angle of the sidewall is another critical parameter. A theoretical relation taking the titling angle into account is compared and in good agreement with experimental results obtained from the trajectory of the floating object. Finally, a continuous motion of the floating object is demonstrated using this control where an array of dielectrowetting electrode pads is sequentially activated.

## 1. Introduction

Surface-tension driven phenomena are ubiquitous in everyday life. For example, when a glass capillary tube is dipped into water, the water level ascends spontaneously inside the tube due to capillary force. Trees utilize this phenomenon to pull water up to the crown. A coin, which has much larger density than that of water, can float on the water surface without sinking, because surface tension contributes to balance the weight of the coin. Small water-dwelling natural creatures, such as water striders, take advantage of surface tension to support their own weight and walk on the water surface. Another interesting example of surface tension is the Cheerios effect, which was coined after observations of cereal flakes floating in milk tending to being attracted to or repelled from the sidewall of the bowl [1,2]. In fact, most of the small floating objects tend to move away from or toward the sidewall. In nature, some water-dwelling creatures harness this phenomenon [3,4]. For example, some small water walkers have developed special tricks by which they change their body to a certain fixed posture and thereby distort the adjacent interfaces. This interface distortion eventually results in generating a net horizontal force on the walkers and automatically climbing the inclined meniscus [4], which is similar to capillary interactions occurring in many small floating objects. In these cases, using typical propelling methods (for example, repeatedly sliding their limbs) is not very efficient for such small water walkers to climb the inclined slippery meniscus forming at the water bank [5].

**Figure 1:**
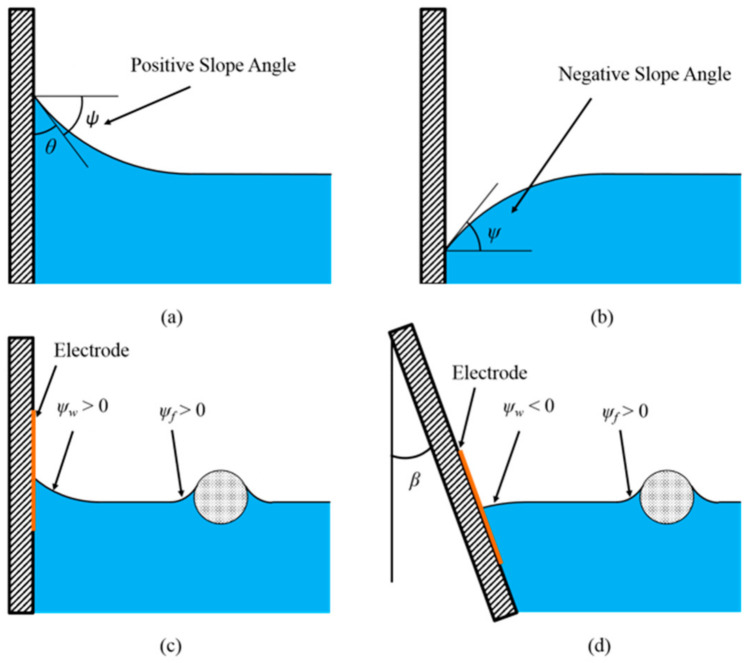
(**a**) Positive slope angle. (**b**) Negative slope angle. (**c**) Dielectrowetting electrodes vertically submerge in dielectric fluid. Due to small surface tension of dielectric liquid, it is difficult to obtain an initially negative slope angle. (**d**) A negative initial slope angle can be obtained by submerging dielectrowetting electrodes in dielectric fluid with a tilting angle β.

The Cheerios effect is generally understood by the lateral component of capillary force [6,7,8]. Both magnitude and direction of this force are determined by many parameters, such as the properties of gas–liquid or liquid–liquid interfaces, the wettability of sidewall, and the properties of floating object. In the early studies, the wettabilities of sidewall and floating object were regarded as the only parameters to determine the capillary interactions, i.e., repulsion or attraction [9,10,11]. However, the recent studies showed that not only the surface wetting properties, but also the densities of floating objects are the critical parameters to determine the net capillary force in the Cheerios effect [12,13,14]. The effects of these multiple parameters can be efficiently dealt by introducing the slope angle ψ that denotes the angle of the interface slope on the wall or floating object with respect to a horizontal line. As shown in Figure 1a, for example, when the slope of the interface is downward (viewed from the object or wall), the slope angle ψ is defined to be positive [12]. On the contrary, as the interface is upward, Figure 1b, the slope angle ψ is negative. The slope angle is generally function of the surface wettabilities, the densities of floating objects, and so on.

A general understanding in the Cheerios effect is that, if the sidewall and the floating object are not very close to each other, they attract each other when the slope angles of the wall and the object have the same signs. On the contrary, they repel when the slope angles of the wall and the object have the opposite signs. The sign of the slope angle of the sidewall is determined by the wettability of the wall surface [7] and the tilting angle of the sidewall [15], while the sign of the slope angle of the floating object depends on the wettability of the object surface and, equally importantly, the density (weight) of the object [7,13]. In particular, the density (weight) of the floating object should be in a certain range for capillary interaction to be effective. As an extreme case, when the floating object is extremely small (for example, the object size is less than 10 µm), the Cheerios effect would not be noticeable, because the weight of the object is too small to create any significant interface distortion [12].

Due to its significance and importance, many attempts have been made to control and utilize the capillary interaction for many applications such as self-assembly processes of two-dimensional array or monolayer on the fluid–fluid interface [16,17,18]. Inspired by meniscus climbing insects, some groups [19,20] mimicked and controlled their climbing principles by using a bent plastic or metal sheet in order to distort the air–liquid interface and climb the inclined meniscus. Our group [13,21] reported an active and electrical control method of capillary interaction with *water* by using electrowetting (more exactly, electrowetting on dielectric or EWOD) to change the wettability of the floating object and/or that of the vertical sidewall. By installing electrowetting electrodes on the floating object or sidewall and applying an electric potential to the electrodes, the air–water interface of the electrodes can be distorted to change the slope angles and thus control the capillary forces between the object and sidewall. By extending this concept, we successfully demonstrated continuous propelling motions of floating objects in a linear path between two vertical walls by sequentially activating arrays of EWOD electrodes. Other groups showed manipulations of partially submerged rods using electrowetting [22]. 

Here, as an extension of electric control of the capillary interaction, the outstanding question may be what if the fluid is not conductive. T = All the above actuations are applicable only for conductive liquids (most commonly water), since the used electrowetting principle works well for conductive liquids but not directly for non-conductive (dielectric) liquids. This article is focused on answering this question. In order to control the capillary interaction with nonconductive (dielectric) liquid, we utilize dielectrowetting (not electrowetting), which is derived from liquid dielectrophoresis and is known to be efficient in changing the wettability of dielectric (nonconductive) liquids. However, unlike the previous work with electrowetting where a vertical wall was used, we found that tilting the sidewall is critical in order to control capillary interaction and to reverse motions between attraction and repulsion. This is due mainly to the fact that most of the dielectric liquids typically have low gas–liquid interfacial tensions. By additionally changing the tilting angle, we were able to successfully control attraction and repulsion between a small object floating on a dielectric liquid and a sidewall. The detailed result and mechanism on this will be presented in the following sections. This paper begins with an experimental study on dielectrowetting. Then, measurements of dynamic interactions (motions) between a floating sphere and a tilted wall are made when dielectrowetting is installed on the tilted wall. These measurements are compared with theoretical calculations, showing a good agreement, especially when the particle and wall are at large separation. Finally, this control of the Cheerios effect is extended to continuously propel small floating objects along a linear path by using two arrays of electrodes. 

## 2. Theoretical Background

Since the present work is mainly focused on the integration of dielectrowetting with the capillary interaction (Cheerios effect), theoretical backgrounds of dielectrowetting and capillary interaction are separately reviewed.

### 2.1. Dielectrowetting

Electrowetting on dielectric (EWOD) has been one of the most versatile and effective methods to actively control the wetting of a solid surface [23,24]. Due to its low power consumption and high reversibility, electrowetting has extensive applications in chemical reactions and biochips [25,26,27], electronic paper [28], tunable-focus liquid lens [29,30], free surface propulsion [13,31], micro-particle manipulation [32,33], displays [34,35], and others. However, although EWOD is highly effective with electrically conductive liquids, it is not very efficient for non-conductive or dielectric liquids. 

On the other hand, in order to actuate and manipulate dielectric liquids, liquid dielectrophoresis (L-DEP) has been developed [36,37,38,39] from particle dielectrophoresis that has been commonly used to separate living and dead cells [40], handle biological cells, or micro particles [41], etc. McHale et al. [42,43] studied L-DEP in perspective of contact angle modulation by localizing the electric field near the contact line. They developed a relation between the contact angle θ and the applied voltage V, in case that liquid is much thicker than the penetration depth of the applied electric field:

(1) $$\cos\theta =\cos\theta_{0}+\frac{\pi \varepsilon_{0}\left(\varepsilon_{l}-1\right)}{8w\gamma_{lv}}{\left(C_{V}V\right)}^{2},$$

where

(2) $$C_{V}=\left(1+\frac{\varepsilon_{d}-\varepsilon_{l}}{\varepsilon_{d}+\varepsilon_{l}}\right)e^{-\pi t_{d}/2w}/\left(1+\left(\frac{\varepsilon_{d}-\varepsilon_{l}}{\varepsilon_{d}+\varepsilon_{l}}\right)e^{-\pi t_{d}/w}\right)$$

is the voltage reduction factor to compensate the voltage loss in the dielectric layer [42], $\theta_{0}$ is the initial contact angle with no voltage applied, $\varepsilon_{0}$ is the vacuum permittivity (8.854 × 10^−12^ F/m), $\varepsilon_{l}$ is the relative permittivity of dielectric liquid, $\varepsilon_{d}$ is the relative permittivity of dielectric layer on the electrode, w is the electrode width or gap between electrodes, $\gamma_{lv}$ is the vapor–liquid interfacial tension, and $t_{d}$ is the thickness of the dielectric layer. Specifically, they coined a new term “dielectrowetting” (electrowetting for dielectric liquids), which is similar to electrowetting in terms of contact angle modulation. Compared to electrowetting, dielectrowetting provides a significant change in contact angle (even superspreading with almost 0° contact angle). By taking advantage of the superspreading, a dielectrowetting optical shutter with dielectric ink droplets has been developed [44,45]. Similar to aqueous (conductive) droplet manipulations on digital microfluidics platforms [24,27], creating, transporting, cutting, and merging of dielectric (nonconductive) droplets have been demonstrated [46]. Reviews on dielectrowetting and its applications for digital microfluidics can be found in the literature [47,48].

**Figure 2:**
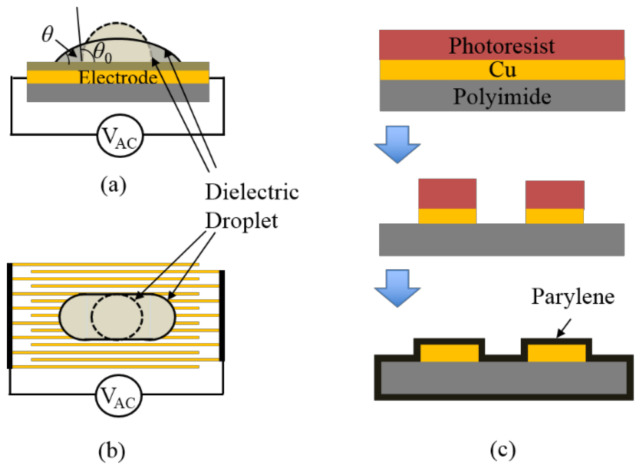
Configuration of dielectrowetting and microfabrication process flow. (**a**) Side view and (**b**) top view of dielectrowetting configuration. Dashed lines are initial droplet shape without electrical voltage applied, while solid lines represent droplet shape after AC signal is applied. (**c**) Microfabrication process flow of dielectrowetting electrodes.

As shown in Figure 2a, the side view of the droplet configuration for dielectrowetting is similar to that for electrowetting Figure 2b in Cho et al. [23]. The major difference is that the dielectrowetting electrodes consist of a set of long interdigitated finger electrodes top view in Figure 2b on a single plate (coplanar), not solid square or rectangular electrode shapes. Strong non-uniform electric fields between the fingers generate L-DEP forces to pull the liquid along the electrodes. Note that the electrowetting electrodes are in general placed on separate plates (e.g., the signal electrode is on the bottom plate, while the ground electrode is on the top plate when a droplet is sandwiched between the two plates). Both electrowetting and dielectrowetting electrodes are covered by a hydrophobic or oleophobic material. 

### 2.2. Cheerios Effect

When a single object stays at an interface of two immiscible fluids, the forces in all direction are balanced: the interfacial tensions are balanced with other forces such as gravitational and buoyant forces. In more detail, the interfaces adjacent to the object would be distorted with a downward or upward slope to provide balancing forces to prevent the object from departing from the interface. When this object approaches another floating object or a stationary wall, capillary forces between the two floating objects or between the floating object and the wall are generated, because the interfaces near the objects and wall are altered and distorted by the interactions between the neighboring objects or wall. The generated capillary forces are typically small (less than tens of µN), so easily buried by random environmental disturbances in many cases, especially for the objects with large mass. In this case, any movement induced by the capillary forces is unnoticeable. However, for micro- or millimeter-sized floating objects, the capillary force comes into play, since the interfacial tension becomes dominant in such scales. As a result, the objects and walls repel or attract each other, for which the horizontal components of the capillary forces are responsible. 

The previous common understanding is that the capillary forces and induced motions depend only on surface wetting properties (i.e., contact angles) [9,10,11], which is not necessarily true for all the scenarios. For objects adjacent to a vertical sidewall where the contact line can move freely without pinning and thus its slope angle ψ remains unchanged, this statement is true. In many situations, however, contact angles are neither the sole parameters anymore nor even sufficient to determine the direction of the capillary forces. Vella and Mahadevan [1] challenged the previous understanding through a simple experiment. By maintaining the similar wettabilities of two floating objects but changing the weight of one object, it was shown that the force between the objects could be reversed, which was well explained by using the slope angle. Thus, recent studies [12,13,14] regarded the slope angles ψ on objects as the most efficient parameter to describe the capillary forces. A better understanding is that they attract each other when the slope angles of the wall and the object have the same signs. In contrast, they repel when the slope angles have the opposite signs. This argument is valid unless the objects are very close to each other.

Suppose that two infinite vertical walls or infinite vertical cylinders are partially submerged in a dielectric liquid (e.g., oil) and interact with each other. An oleophilic surface of them results in a positive slope angle, while an oleophobic surface leads to a negative slope angle. That is, the slope angles are only determined by the contact angles θ. However, when a partially submerged infinite wall is tilted with an angle of β, the slope angle is determined by the contact angle as well as the tilting angle β (Equation (7)). An oleophilic surface does not always have a positive slope angle, because the tilting angle of the wall also has an effect on the slope angle. For the oleophobic surface, the same argument can be applied. In the meantime, for a floating object with vertical side surfaces, the contact angle is still the only parameter to determine the slope angle as long as the contact line can freely move on the side surfaces. However, when the density of the object is very large or small compared to that of the liquid, the contact line may be pinned on the upper or lower edge of the object and eventually provide an extra force to prevent it from sinking or arising. In this case, the contact angle becomes a meaningless parameter. When an object with an arbitrary shape floats at the interface, this mechanism becomes more complicated.

For the sake of simplicity, let us consider a case in which a sphere with radius R and density ρ floats at the liquid–gas interface. Due to the curved surface of the sphere, the slope angle varies as the contact line is located at a different location on the sphere surface, even though the contact angle remains unchanged. Yuan et al. [13] reported that the density and surface wettability of the floating sphere as well as the density and interfacial tension of the fluid affect the slope angle of the floating sphere and thus the capillary forces. 

The horizontal component of the capillary force between a floating sphere and a vertical sidewall with a fixed contact angle can be calculated by solving the linearized Young–Laplace equation [7]:

(3) $$F_{x}=-\pi \gamma_{\mathrm{lv}}\left[2qQ^{2}K_{1}\left(2qs\right)+2QqHe^{-qs}+q{\left(rqHe^{-qs}\right)}^{2}\right]$$

where

(4) $$q^{-1}=L_{c}\approx \sqrt{\gamma_{lv}/\rho_{l}g}$$

is the capillary length, g the gravitational acceleration, Q the capillary charge of the sphere, r the radius of the contact line on the sphere surface, $K_{1}$ the modified Bessel function, $\rho_{l}$ is the density of fluid, $\gamma_{lv}$ is the gas–liquid interfacial tension, and s the separation between the center of the sphere and the vertical wall. For a floating sphere,

(5) $$Q=r\sin\psi_{f}$$

where subscripts f and w represent the floating object and wall, respectively. H in Equation (3), which is the contact line elevation on the vertical wall for a given contact angle $\theta_{w}$, can be expressed as follows [11]:

(6) $$H=\frac{\sin\psi_{w}}{\left|\sin\psi_{w}\right|}L_{c}\sqrt{2\left(1-\cos\psi_{w}\right)}$$

For the vertical wall, $\psi_{w}=90^{{}^{\circ}}-\theta_{w}$, so H is positive for the oleophilic surface ($\theta_{w}<90^{{}^{\circ}}$) and is negative for the oleophobic surface ($\theta_{w}>90^{{}^{\circ}}$). 

At a moderate or large separation s, the first term and third term in Equation (3) are much smaller than the second term [13]. As a result, unless the separation is too small, Equation (3) can be simplified to:

(7) $$F_{x}\approx -2\pi \gamma_{\mathrm{lv}}QqHe^{-qs}$$

Equations (4)–(6) reveal that for the moderate or large separation s, the force between a floating sphere and a vertical wall is attractive when $\psi_{f}$ and $\psi_{w}$ have the same signs, and it is repulsive when they have the opposite signs.

As studied in Yuan et al. [13,21], electrowetting can reversibly switch the surface wettability between hydrophobic and hydrophilic states for conductive liquid and thus can change the slope angles. In the previous study, an electrowetting electrode was installed either on the floating object or on the vertical sidewall Figure 1c. When a DC electrowetting signal was applied, the initial hydrophobic surface of the electrode becomes hydrophilic, and the contact line moves upward. As a result, the initial negative slope angle is changed to be positive, and the horizontal component of capillary force is reversed. Thus, electrowetting could control the capillary force direction and magnitude.

The similar control concept can be applied for a non-conductive (dielectric) fluid. However, electrowetting should be replaced by dielectrowetting, since non-conductive fluids are more effectively actuated by dielectrowetting. An additional issue with dielectric fluids is the initial contact angle on the electrode surface is usually less than 90°, even on a Teflon surface, since most of the dielectric liquids typically have low gas–liquid interfacial tensions, which results in a positive slope angle on a vertical wall. In this case, when an AC signal is applied to the dielectrowetting electrodes, although the contact angle further decreases, the slope angle remains positive. However, in order to switch the direction of the capillary force, the slope angle needs to change its sign. As a result, the configuration shown in Figure 1c is not effective for the present control method with dielectric fluids. Initially, the slope angle on the wall should be negative. This initial condition can be achieved by tilting the sidewall with an angle of β, as shown in Figure 1d, where the slope angle becomes:

(8) $$\psi_{w}=90^{{}^{\circ}}-\beta -\theta_{w}$$

Even in case that the contact angle $\theta_{w}$ is less than 90°, the slope angle $\psi_{w}$ can be negative by adequately choosing the tilting angle β. In this case, when a dielectrowetting signal is applied, the contact angle $\theta_{w}$ decreases, which can cause the slope angle to be positive and thus the capillary force to be reversed. 

**Figure 3:**
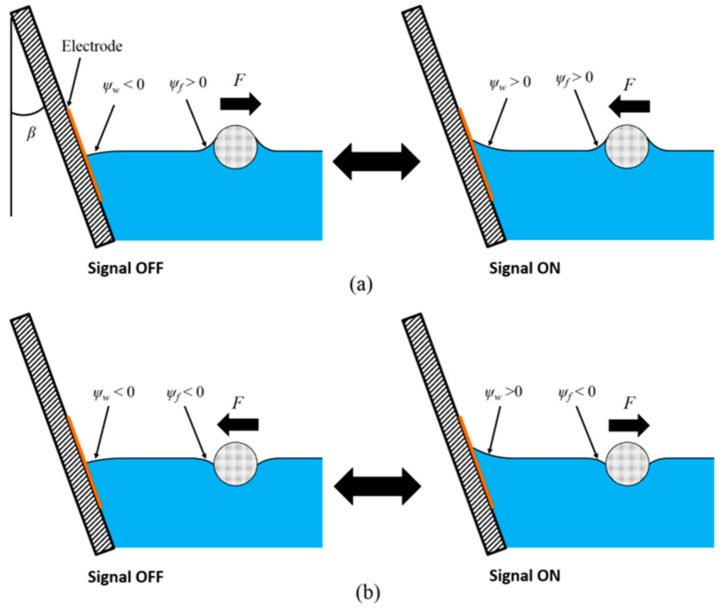
Control concept of Cheerios effect by dielectrowetting. (**a**) The floating object has a positive slope angle; (**b**) the floating object has a negative slope angle.

Figure 3 shows two configurations for dielectrowetting control. Depending on the initial contact angle of the dielectric liquid, the tilting angle can be properly chosen to make the initial slope angle negative. When the applied signal is off, the contact angle is initially large, so the slope angle at the wall is negative ($\psi_{w}$ < 0). As a result, the wall would repel the object of a positive slope angle, $\psi_{f}$ > 0, left in Figure 3a, because the signs of $\psi_{w}$ and $\psi_{f}$ are opposite. When the object slope angle is negative, $\psi_{f}$ < 0, left in Figure 3b, the wall would attract the object, because the signs of $\psi_{w}$ and $\psi_{f}$ are the same. When the signal is turned on, the contact angle reduces, which results in a positive slope angle ($\psi_{w}$ > 0) at the wall. Therefore, the wall would attract the object of the positive slope angle $\psi_{f}$ > 0, right in Figure 3a and repel the object of the negative slope angle $\psi_{f}$ < 0, right in Figure 3b. Consequently, the capillary force in each case would be reversed. These two control configurations are experimentally studied in the following sections.

## 3. Experimental Result

### 3.1. Dielectrowetting

Figure 2c shows the microfabrication process for the present dielectrowetting electrodes. A thin, flexible sheet with a Cu layer of 9 μm in thickness (DuPont Pyralux^®^ flexible Cu product) is patterned by the standard lithographic process and wet-etched by an aqueous FeCl_3_ solution. Both width of and spacing between interdigitated electrodes are equal (50 or 75 µm). Then, a 2.5 µm thick Parylene layer is coated on the top as the dielectric layer. Finally, a thin Teflon layer is dip-coated on the top to make the final surface less oleophilic. In order to minimize viscous friction against floating object motions, the testing fluid is preferred to have low viscosity. Propylene carbonate, a dielectric liquid with viscosity about 20 times lower than that of 1,2 propylene glycol used in McHale’s group [42,43], is selected for the dielectric testing fluid. 

**Figure 4:**
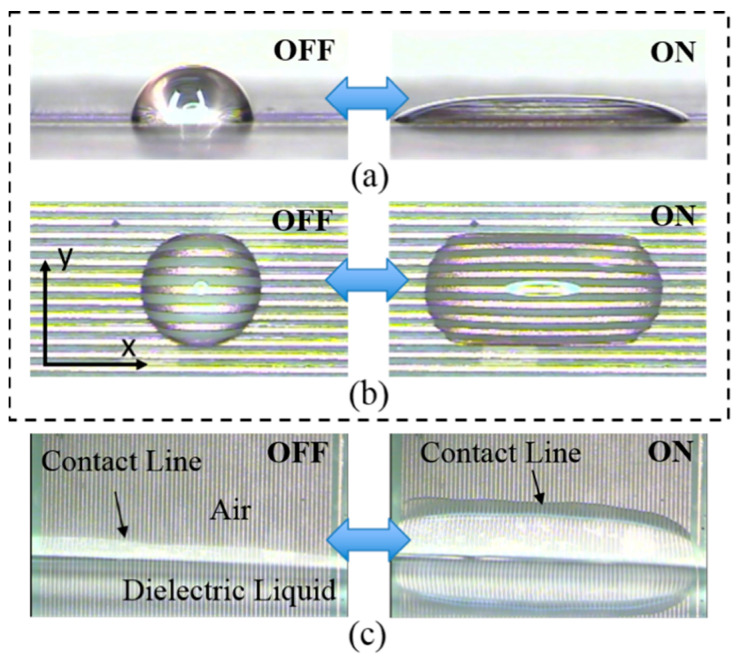
Experimental snapshots of dielectrowetting. (**a**) Side view and (**b**) top view of dielectrowetting droplet modulation; (**c**) contact line climbs vertically submerged dielectrowetting electrodes (V = 300 V_rms_, f = 10 kHz). Movie clips are available in the supplementary information (Movie1, Movie2, and Movie3).

Dielectrowetting is not effective for DC or low frequency AC voltage due to the high impedance of the dielectric layer plus the dielectric droplet. However, when a high-frequency voltage (>kHz) is applied, the contact angle of the dielectric droplet significantly reduces (Figure 4a,b, movie clips, Movie1 and Movie2, are available in the supplementary information), in some cases even reaching superspreading of the droplet. As the input voltage is removed, the droplet returns to its initial shape. As shown in Figure 4b, in contrast to the axisymmetric droplet spreading of electrowetting, the contact angle does not significantly vary in the lateral plane (minor axis), but the major contact angle change occurs in the axial plane along the long finger electrodes (major axis) when dielectrowetting is on.

**Figure 5:**
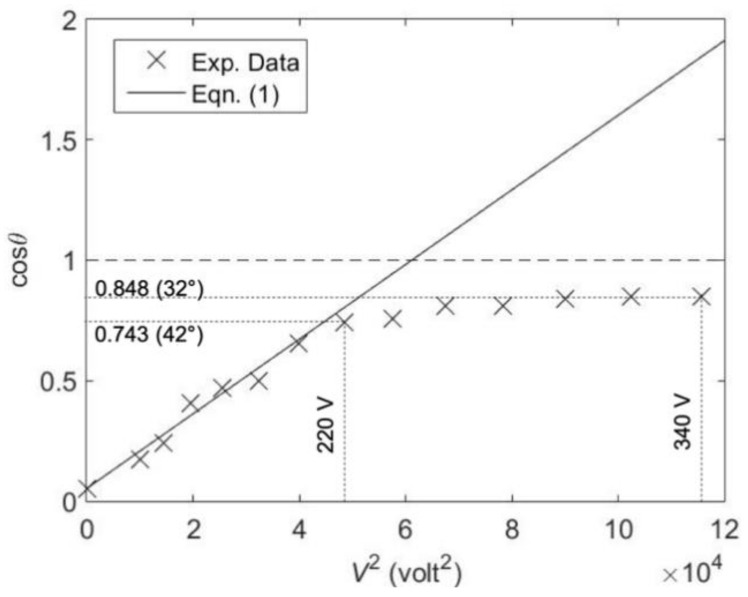
Theoretical and experimental comparison of cosine of contact angle versus the square of voltage (f = 10 kHz).

Figure 5 shows a comparison between the theoretical prediction Equation (1) and the experimental data of cosine of contact angle versus the square of voltage. The width of and the spacing between electrodes are 50 µm. The contact angle was measured by using the side images of the droplet at the contact line. It decreases from 87° to 32° when the applied voltage (10 kHz) increases from 0 to 340 V_rms_. A slightly larger contact angle change from 96° to 23° was recorded by the other group by using the different dielectric layer and dielectric fluid [42]. The droplet spreads much wider and the height of the droplet apex becomes much smaller than electrowetting cases. For the high applied voltage (340 V_RMS_ in this experiment), the droplet becomes unstable and splits into small droplets. In the calculation, the dielectric constants of parylene layer and propylene carbonate are 3.1 and 64, respectively. The interfacial tension of propylene carbonate with air is 41 mN/m. The voltage reduction factor $C_{V}$ is calculated to be 0.38, which is much smaller than the 0.6–0.8 reported previously [42] because of the thicker dielectric layer used in this experiment and greater relative permittivity of dielectric liquid. At low voltages, the theoretical prediction and the experimental data match well. However, at high voltage starting from about 220 V_rms_, the contact angle starts to deviate from the theoretical curve, which is similar to the contact angle saturation of electrowetting. As of today, the saturation of contact angle in electrowetting as well as dielectrowetting is not clearly understood, although many attempts have been made to study it [24]. 

When the dielectrowetting electrode pad with a set of interdigitated electrodes vertically and partially submerge in the dielectric liquid, the contact line quickly climbs up upon dielectrowetting activation Figure 4c; movie clip Movie3 is in the supplementary information. Because the change in the contact angle for dielectrowetting is larger (typically larger than 45°) than that of electrowetting (less than 40°), the climbing height of the contact line is also higher than that of electrowetting.

### 3.2. Control of Cheerios Effect by Dielectrowetting

**Figure 6:**
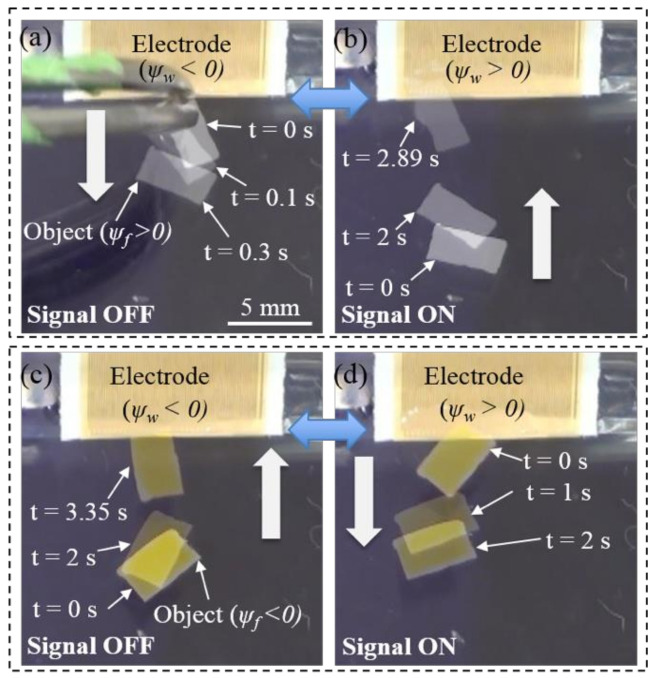
Snapshots of floating object motions controlled by dielectrowetting. Initial repulsion (**a**) of object (positive $\psi_{f}$) is switched to attraction (**b**) by dielectrowetting. Initial attraction (**c**) of object (negative $\psi_{f}$) is switched to repulsion (**d**) by dielectrowetting. Top views, β = 30°, V = 300 V_rms_, f = 10 kHz. Movie clips (Movie4 and Movie5) are available in the supplementary information).

Experimental verifications of the present control scheme Figure 3 are shown in Figure 6 (movie clips, Movie4 and Movie5, are available in the supplementary information). The floating objects used in this experiment are flat thin pieces of plastic with different densities. The slope angle of the floating object is determined not by the wettability of the object material, but by the object density, because the contact line pins at the top or bottom edges of the object. The width of and the spacing between electrodes are 75 µm. When the dielectrowetting signal is off, a low-density floating object (ρ ≈ 0.1 g/cm^3^) with the positive slope angle $\psi_{f}$ (6°) is initially repelled by the tilted sidewall (β = 30°) that has negative $\psi_{w}$ −25°, Figure 6a. When the dielectrowetting signal (V = 300 V_rms_, f = 10 kHz) is turned on Figure 6b, the contact angle reduces and the interface slope $\psi_{w}$ becomes positive. As a result, the capillary force is changed to be attractive. On the contrary, when a high-density floating object (ρ ≈ 1.5 g/cm^3^) is used, the initial slope angle $\psi_{f}$ is negative (−11°). The initial attraction Figure 6c is switched to repulsion Figure 6d by dielectrowetting (V = 300 V_rms_, f = 10 kHz). All these switching operations are reversible. 

The 30° wall tilting angle was carefully picked with the following considerations. Because the initial contact angle is 85°, the tilting angle has to be greater than 5° to achieve an initial negative slope on the wall angle according to Equation (7). At the same time, the contact angle decreases to 32° with the voltage turns on, which means that the tilting angle needs to be less than 58° to have a positive slope angle with the electric signal on. As a result, the theoretical tilting angle for this floating object manipulation experiment should range between 5° and 58°. In addition, if the tilting angle is very close to the upper or lower limit, the resulted slope angle would be very close to zero, which would not generate sufficient free surface distortion and thus capillary force to attract or repel the floating object. As a result, 30° tilting angle, which is about the midpoint, was selected for the above experiment. 

Since the capillary force is very small (typically less than tens of µN), its direct measurement is not easy. Nevertheless, some of the efforts have already been made to verify Equation (3). Dushkin et al. [49] used torsion balance to directly measure the horizontal component of capillary force. However, their results had large errors for small separation, even though they were in good agreement for large separation. Yuan et al. [13] indirectly verified Equation (3) by two methods: First, inspired by another report [50], they measured and compared the critical contact angles where there is a zero force between the floating sphere and the vertical sidewall. The electrowetting principle allows them to continuously change the contact angle of the wall to find the critical contact angle. Second, they measured the dynamic motion of the floating sphere under electrowetting actuation. The obtained distance–time relationship was verified against the theory. 

**Figure 7:**
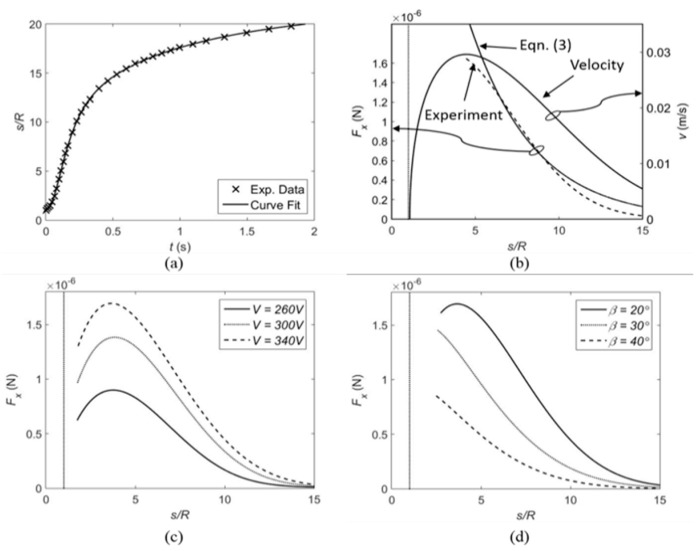
Comparison between Equation (3) and experiment and parameter effects. (**a**) The displacement of the floating sphere is measured by high-speed images when AC signal is applied. (**b**) The velocity of the particle is obtained by taking the first derivative of the measured displacement. The horizontal component of capillary force is obtained by substituting measured velocity into Equations (8) and (9) and is compared with theory Equation (3). (**c**) Effect of applied voltage (*f* = 10 kHz, β = 40°); (**d**) Effect of tilting angle (*V* = 340 V_rms_, *f* = 10 kHz). The thin dotted vertical lines denote the smallest separation possible between the sphere and wall.

In this paper, we use the motion measurement of the floating sphere to compare the experimental data with Equation (3). A dielectrowetting electrode pad with interdigitated electrode width and pitch of 75 µm is submerged in propylene carbonate liquid with a tilting angle β = 20°. The initial contact angle is 85°, so the slope angle is −15° according to Equation (7). A glass sphere (R = 0.5 mm, ρ = 2.5 g/cm^3^) is coated by Teflon to make the contact angle higher ($\theta_{f}$ ≈ 85°). The slope angle $\psi_{f}$ (≈ −6.7°) is calculated by using iteration method [8]. The floating sphere is initially in contact with the electrode pad by the capillary force due to the initial negative slope angles of the sphere and the wall. When a dielectrowetting signal (V = 340 V_rms_, f = 10 kHz) is applied, the contact angle on the electrodes reduces to 32° and the wall slope angle $\psi_{w}$ also changes to +38°. As a result, the wall starts to repel the sphere. The dynamic motion of the sphere is recorded by a high-speed camera. The displacement of the sphere is digitized in each time frame and is curve-fitted to a rational polynomial, as shown in Figure 7a. The velocity v and acceleration a are obtained by taking the first time-derivative and second time-derivative of the displacement, respectively. The velocity of the sphere is shown in Figure 7b. The capillary repulsive force initially accelerates the sphere. As the distance between the sphere and the wall increases, the capillary force becomes weaker, while the drag force becomes relatively larger because of the increased velocity. As the sphere further moves away from the wall, the sphere decelerates due to weak capillary force and dominant drag force. 

For this dynamic motion, the force balance [51] in the horizontal direction is given as,

(9) $$F_{x}=ma+F_{drag},$$

where m, $F_{drag},$ and $F_{x}$ are the mass of the sphere, the drag force, and the horizontal component of capillary force acting on the sphere. The Stokes drag expression used in the references [51,52] may not be applicable, because the Reynolds number is not too small (>20) and the sphere is partially submerged. Therefore, the following expression (more general for moderate Reynolds numbers and the partially submerged configuration) is used to describe the present drag [53]:

(10) $$F_{drag}=\frac{1}{2}C_{d}\rho_{l}v^{2}A$$

where $C_{d}$ is the drag coefficient and A is the projection area of the submerged part of the sphere. By substituting v and a into Equations (8) and (9), the capillary force $F_{x}$ on the sphere can be calculated and compared with Equation (3). In this calculation, $C_{d}$ is assumed to be 4. As shown in Figure 7b, the experimental result is in good agreement with Equation (3) in the range of intermediate separation (*s/R* = 6–10). At small separation, the theory overestimates the force. This may be due to the large distortion of the interface that breaks the assumption made in the theory based on the linearized Young–Laplace equation. In addition, the initial sphere movement may be affected by the surface wave generated by the moving contact line while the signal is turned on. In comparison with Figure 6 in Reference 13 that was obtained by the similar method to determine the capillary force between floating particle and electrowetting controlled vertical wall, the forces at the same separation distance are very similar to each other, although the surface distortion at the wall is much larger with dielectrowetting. This is due possibly to the lower surface tension of the dielectric liquid. Because of the higher viscosity of the dielectric fluid used in this experiment, the peak velocity with dielectrowetting is slower than that with electrowetting, but the velocity reaches its peak when the floating object is closer to the wall compared to the electrowetting experiment.

Figure 7c,d shows the effects of applied AC voltage and the wall tilting angle on the lateral capillary force obtained by the dynamic motion method. When the signal frequency is fixed at 10 kHz and the applied voltage increases while the tilting angle β is maintained at 20° Figure 7c, the lateral force increases. It is easy to understand, because the change in the contact angle is larger at higher voltages. The effect of the wall tilting angle is shown in Figure 7d. When the applied voltage is fixed at 340 V_rms_, the lateral force decreases as the tilting angle increases. According to Equation (7), changing the tilting angle alters the slope angles. The smaller tilting angle results in the larger wall slope angle as well as larger force. 

**Figure 8:**
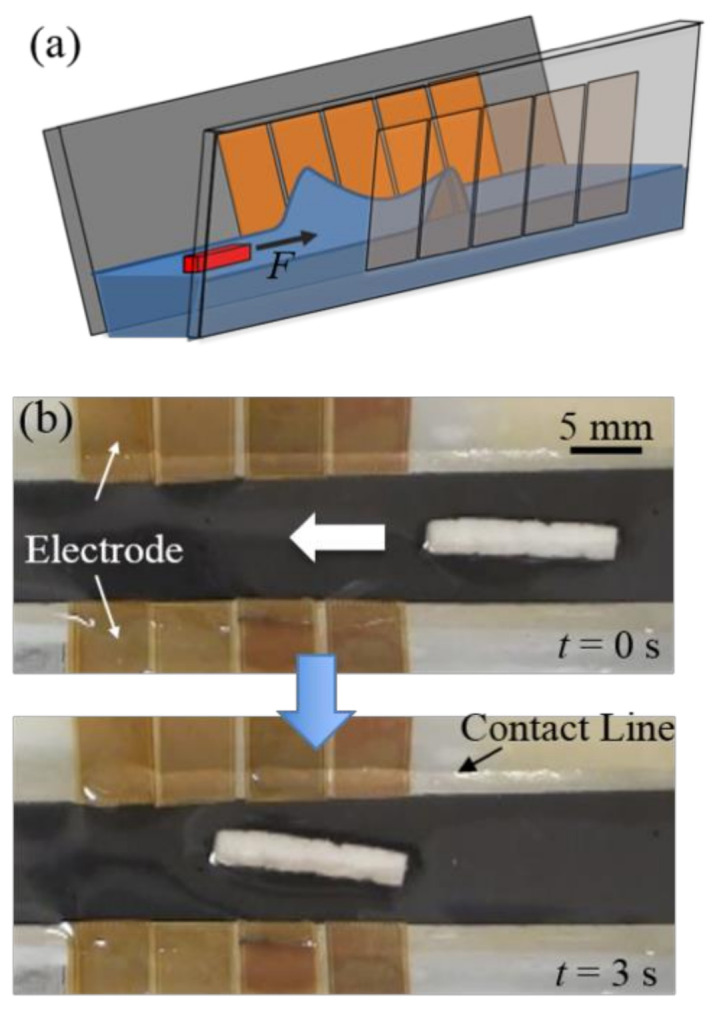
(**a**) Continuous propulsion concept; (**b**) top view snapshots of continuous transporting in channel. Pairs of electrode pads are activated sequentially from right to left with duration of 0.7 s (V = 300 V_rms_, f = 10 kHz, β = 30°). Movie clip (Movie6) is available in the supplementary information.

When simply turning dielectrowetting on or off with a single electrode pad, the motion of floating objects is limited to the space between the wall and objects (one-time movement toward or far away from the sidewall). Figure 8a illustrates how to continuously transport a floating object in a channel by dielectrowetting. Unlike the vertical sidewalls used in the electrowetting case, here the sidewall should be also tilted with a tilting angle to obtain the initial negative slope angles. Two arrays of electrode pads are attached to two tilted sidewalls. $\psi_{w}$ is initially negative, so an object with positive $\psi_{f}$ automatically stays in the middle of channel due to symmetric repulsion from both sidewalls. When a pair of electrode pads (one on each side wall) is activated together, the slope angles $\psi_{w}$ converts from negative to positive angles. As a result, the interface in front of the floating object rises and generates a pulling force on the floating object. Due to symmetry, the lateral forces on the object exerted by the two sidewalls are cancelled out. Only a net force on the object is toward the elevated interface (along the channel), so the object is propelled along the channel path. By shifting the activation to the next pair, a continuous movement is achieved.

Figure 8b shows a low-density object ($\psi_{f}$ > 0) is propelled in the channel with two arrays of dielectrowetting electrode pads (5 mm width) (a movie clip, Movie6, is available in the supplementary information). Upon shifting activations of electrode pad pairs to the left (V = 300 V_rms_, f = 10 kHz) for a duration of 0.7 s, the object is step-by-step propelled to the left. A sequential digital signal is provided by a microcontroller (ATMEL ATtiny24A). Relays (Panasonic AQW614EH) are used for transmitting a high AC voltage from the amplifier to the electrodes. 

It was observed that the object was sometimes pulled toward one side of the channel (shown at Movie6). Ideally, the lateral forces from the two sidewalls should be cancelled out. However, a small deviation of the floating object from the center line and/or a small free surface distortion difference resulted from different contact angles or wall tilting angles could generate an asymmetric resultant force. 

## 4. Concluding Remarks and Future Works

The main goal of this article is to study how to electrically control the capillary interaction between the sidewall and objects floating on a dielectric (nonconductive) liquid. The key idea is to implement *dielectrowetting* to control the capillary interaction. By electrically changing the wettability of the dielectric fluid and then reversing the sign of the slope angle near the side wall, attraction and repulsion of the floating object with respect to the sidewall is controlled in the on-demand manner. Here, the tilting angle of the sidewall is found to be critical for switching between attraction and repulsion because of the low surface tension of the dielectric liquid. When dielectrowetting is applied, the dynamic motion of the floating sphere is recorded and analyzed. The experimentally obtained force is compared with a theoretical calculation and shows a good agreement when separation between the object and wall is not small. The deviation at small separation may be caused by the non-linear effect resulting from large displacements of the interface on the dielectrowetting electrodes. Finally, a continuous linear motion of the floating object is achieved using an array of dielectrowetting electrode pads. By sequentially shifting activations of arrayed electrode pads, linear translations of floating objects in the small channel are demonstrated.

For the future application, the developed control method may be applied to assemble micro- or mini-floating objects to build more complex structures. In a theoretical perspective, the present theory can be further developed to find solutions or models, even when the distance between the objects is small, the distortion is large, and/or the shape of floating object is more complex. In this case, the linearization of Young–Laplace equation would be short-handed, but other theoretical frameworks [54,55] may be applicable. 

## Supplementary Materials

The following are available online at https://www.mdpi.com/2072-666X/12/3/341/s1, Movie1, Movie2, Movie3, Movie4, Movie5, and Movie6.
